# Higher methylation intensity induced by EBV LMP1 via NF-κB/DNMT3b signaling contributes to silencing of PTEN gene

**DOI:** 10.18632/oncotarget.9474

**Published:** 2016-05-19

**Authors:** Hong Peng, Yuxiang Chen, Pinggui Gong, Longmei Cai, Xiaoming Lyu, Qiang Jiang, Jianguo Wang, Juan Lu, Kaitai Yao, Kunping Liu, Jinbang Li, Xin Li

**Affiliations:** ^1^ Department of Otorhinolaryngology at Nanfang Hospital, Cancer Research Institute, Southern Medical University, Guangzhou 510515, China; ^2^ Department of Otolaryngology-Head and Neck Surgery, the Second People's Hospital of Guangdong Province, Southern Medical University, Guangzhou 510515, China; ^3^ Department of Pathology, the Sixth Affiliated Hospital of Guangzhou Medical University, Qingyuan 511518, China

**Keywords:** PTEN, DNMT3b, DNA methylation, EBV, LMP1

## Abstract

Phosphatase and tensin homolog (PTEN) is a major tumor suppressor and usually silenced via the deletion, insertion and mutation. We previously discovered its inactivation via aberrant CpG island methylation. Here, we provide further evidence that EBV latent membrane protein 1(LMP1) can induce a higher intensity of DNA methylation at PTEN CpG islands, inactivating PTEN at the cellular and molecular level. Initially, increased methylation intensity of PTEN CpG islands was observed in EBV-infected nasopharyngeal carcinoma (NPC) cells, accompanied by decreased PTEN expression. In NPC tissue samples showing the methylation at PTEN promoter, LMP1 was highly expressed in higher methylation intensity group relative to lower intensity group, and DNA methyltransferase 3b (DNMT3b) expression was positively correlated with LMP1 expression. Moreover, transfection of LMP1 gene into EBV-negative NPC cells demonstrated that LMP1 up-regulated DNMT3b expression, leading to a higher intensity of PTEN CpG island methylation. Mechanistically, computational prediction and luciferase reporter assay identified a functional NF-κB binding site on DNMT3b promoter and the mutated NF-κB binding site abolished LMP1-mediated DNMT3b activation. Chromatin immunoprecipitation displayed that NF-κB p65 subunit constitutively bound to DNMT3b promoter, supporting the activation of DNMT3b by EBV LMP1 via NF-κB signaling. Furthermore, the expression level of DNMT3b was observed to be increased in the nuclei of LMP1-expressing NPC cells, and a NF-κB inhibitor, PDTC, counteracted LMP1-mediated DNMT3b overexpression. Thus, this study first reports that LMP1-mediated NF-κB can up-regulate DNMT3b transcription, thereby leading to relatively higher methylation intensity at PTEN CpG islands, and ultimately silencing major tumor suppressor PTEN.

## INTRODUCTION

PTEN is a major tumor suppressor gene (TSG) commonly inactivated in various cancers, being implicated in many aspects of the malignant phenotypes, such as proliferation, transformation, invasion, and metastasis [1–4]. Silencing of TSGs via deletion, insertion and mutation is an uncommon event in NPC carcinogenesis and aberrant methylation has been emerging as an alternative mechanism [5–9]. We previously discovered the effect of aberrant CpG island methylation on PTEN inactivation in NPC [5], but its underlying mechanism still remains unclear.

Epstein-Barr virus (EBV) infection has been long believed to be associated with many malignancies including Burkitt's lymphoma, Hodgkin lymphoma, gastric cancer and NPC. Its infection belongs to the latency II program in NPC, and is typically restricted to express several transcripts, such as EBV-determined nuclear antigen 1 (EBNA1), EBER, latent membrane protein 2A (LMP2A), LMP1, and transcripts from the BamHI A region (BARF0) [10]. As an important hallmark for the latency program of EBV infection, EBV LMP1 has the ability to transform rodent cells and render cell growth in soft agar [11]. In particular, LMP1 can augment DNA methyltransferases (DNMTs) that impel the aberrant methylation of many host genes, including TSG, through some signaling pathways, such as the c-Jun NH2-terminal kinase-activator protein-1 [12] and the Rb-E2F signaling [13, 14]. Currently, the role of NF-κB in LMP1-dependent regulations is perceived as extremely important. Enhanced NF-κB signaling is a critical facet of LMP1-associated signaling [15]. Many phenotypic changes such as cell survival, oncogenesis control and cancer therapy resistance are attributable to LMP1′s ability to activate NF-κB [15–17]. These findings give a hypothesis that LMP1 may promote the aberrant methylation at PTEN CpG islands probably by means of some similar regulatory mechanism.

Therefore, the aim of the present study is to clarify how EBV-encoded LMP1 can epigenetically alter the expression of host major TSG PTEN. We provided cellular and clinical evidence revealing a close correlation among LMP1 expression, DNMT3b expression, and the methylation intensity at PTEN CpG islands, and further disclosed that LMP1-mediated NF-κB up-regulated DNMT3b transcription by binding to DNMT3b promoter, leading to the higher methylation intensity at PTEN CpG islands and ultimately silencing tumor suppressor PTEN in NPC.

## RESULTS

### Silenced PTEN expression is relevant to the higher PTEN methylation intensity in EBV-infected NPC cells

**Figure 1:**
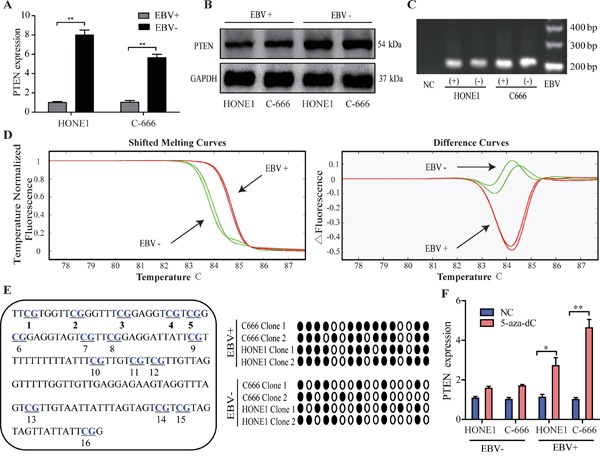
Down-regulated PTEN and higher CpG island methylation intensity in NPC cell lines with EBV infection **A.** The expression of PTEN mRNA in EBV-positive and EBV-negative NPC cells. **B.** The expression of PTEN protein in EBV-positive and EBV-negative NPC cells. **C.** The detection of methylation status of PTEN in EBV-positive and EBV-negative NPC cells. MSP analysis was performed using primers specifically for the methylated CpG sites of PTEN gene. PCR products were visualized after electrophoresis on a 2% agarose gel. **D.** HRM results of the EBV-positive and EBV-negative NPC cells. Melting curves of the EBV-positive and EBV-negative NPC cells as well as the corresponding difference curves are shown. The grouping is as calculated by the LightScanner software. **E.** Sequencing analysis of PCR products of MS-HRM for PTEN in EBV-positive and EBV-negative NPC cells. Left, the location of 16 CpG sites in PTEN CpG island. Right, four clones picked from PCR products of MS-HRM for PTEN in EBV-positive and EBV-negative NPC cells were sequenced, respectively. Open circles, unmethylated CpG; closed circles, methylated CpG. **F.** The expression of PTEN mRNA in EBV-positive and EBV-negative NPC cells incubated with either DNA methylation inhibitor 5-Aza or control. mRNA abundance was normalized to GAPDH mRNA. Each bar represents the means ± SD of three experiments, **p<0.01.

To determine the possible link between EBV infection and PTEN expression, we examined PTEN expression level in EBV-positive and EBV-negative NPC cells. Notably, PTEN mRNA and protein were significantly down-expressed in EBV-positive NPC cells compared with EBV-negative NPC cells (Figure 1A, 1B), implying the possible relevance of EBV infection to silenced PTEN expression.

We next compared the methylation at PTEN CpG islands between EBV-positive and EBV-negative NPC cells. As the 5′ promoter region of PTEN contained many CpG islands spanning_~_ 3 kb, we focused our attention upon the densest region reported in our previous study [1]. Initially, MSP assay, a conventional method for testing of methylation status, showed that the CpG islands of PTEN promoter were methylated in both EBV-positive and EBV-negative NPC cells (Figure 1C). Subsequently, MS-HRM assay, a higher sensitive and reproducible approach for the quantitative methylation detection [18, 19], was conducted to assess the methylation intensity at PTEN CpG islands in EBV-positive and EBV-negative NPC cells (Figure 1D). Impressively, the shifted melting curves of MS-HRM products amplified from EBV-positive NPC cells were obviously higher than that of EBV-negative cells. To confirm the reliability of MS-HRM results, we sequenced the PCR products amplified from NPC cells using MS-HRM primers of PTEN. Consistently, the methylation frequency of CpG sites within this region was much higher in EBV-positive NPC cells than that in EBV-negative NPC cells (Figure 1E).

Furthermore, EBV positive or negative NPC cells were incubated with a DNA methylation inhibitor 5-Aza (5-aza-2′-deoxycytidine). 5-Aza obviously restored PTEN expression to a higher extent in EBV-positive cells than EBV-negative cells (Figure 1F).

Collectively, these in vitro results suggest that EBV infection is associated with the methylation intensity at PTEN CpG islands, probably contributing to the silencing of PTEN expression.

### The higher PTEN methylation intensity is attributed to EBVLMP1

**Figure 2:**
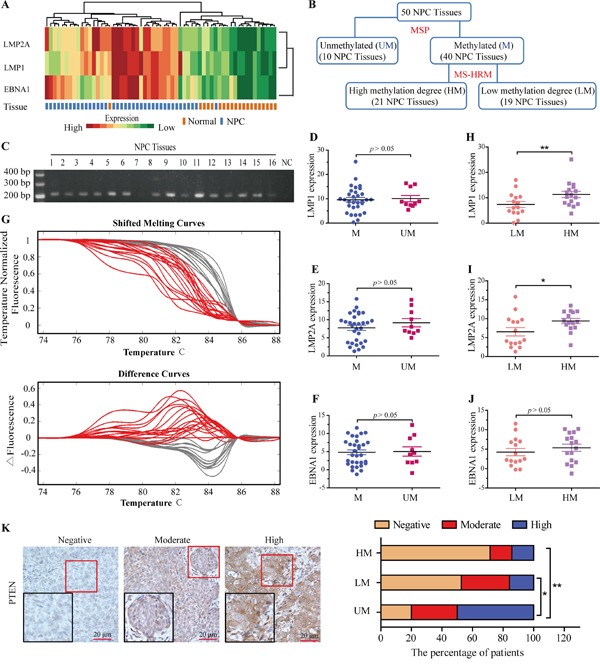
Higher methylation intensity of PTEN CpG islands in NPC specimens with EBV infection **A.** Hierarchical clustering of the EBNA1, LMP2A, LMP1 expression levels was generated by R programming language based on quantitative PCR results in NPC and NP samples. High and low expressed genes are shown by red and green, respectively. **B.** NPC tissues were divided into methylated (M) and unmethylated (UM) groups based on the MSP analysis results, and then the methylated group was subdivided into the high methylation intensity (HM) and low methylation intensity (LM) groups based on the MS-HRM analysis. **C.** Detection of methylation status of PTEN in NPC samples. **D-F.** Average expression levels of LMP1, LMP2A and EBNA1 in M and UM groups based on MSP analysis. **G.** MS-HRM results of the 40 methylated NPC tissues. Melting curves of the methylated NPC tissues as well as the corresponding difference curves are shown. The grouping is as calculated by the LightScanner software. **H-J.** Average expression levels of LMP1, LMP2A and EBNA1 in HM and LM group. mRNA abundance was normalized to GAPDH mRNA. **K.** Immunohistochemistry detection of PTEN protein in patients with NPC. Left, representative IHC of NPC samples, showing the negative, moderate and high expression level of PTEN, respectively. Right, the percentage of patients with the negative, moderate and high expression of PTEN protein in UM, LM and HM groups. Each bar represents the means ± SD of three experiments. *p<0.05, **p<0.01.

To replicate the in vitro results, we in vivo evaluated the relation of EBV infection with PTEN methylation in clinical tissue samples. It was known that EBV consistently occurred in NPC cells and is restricted to express several transcripts [10], so we examined the expression levels of EBNA1, LMP2A, and LMP1 by qPCR in 50 NPC specimens and 22 NP tissue samples. As expected, the average expression levels of EBNA1, LMP2A, and LMP1 were obviously higher in NPC specimens than in NP tissues (Figure 2A).

We next performed conventional MSP analysis to examine the methylation at the CpG islands of PTEN promoter in these NPC specimens and NP tissue samples. The CpG islands of PTEN promoter were methylated in 80% (40/50) of NPC tissues (Figure 2B, 2C). Similar to in vitro results, it is in all NPC specimens that there was no significant difference in the expression levels of EBNA1, LMP2A, and LMP1 between 40 methylated (M) and 10 unmethylated (UM) NPC tissue samples (Figure 2B, 2D-2F).

Alternatively, using MS-HRM analysis, we quantitated the intensity of DNA methylation at CpG islands of PTEN promoter in 40 NPC tissues that harbored the methylated PTEN promoter. We divided these NPC samples into the high methylation intensity(HM) and low methylation intensity (LM) groups according to MS-HRM results (Figure 2B, 2G), and then compared the expression levels of EBNA1, LMP2A and LMP1 between these two groups. Notably, the average expression levels of LMP1 (11.31±1.240, *P* = 0.0183) and LMP2A (9.39±0.698, *P* = 0.0371) but not EBNA1 were significantly higher in HM group than in LM group (Figure 2H-2J). Association between PTEN methylation and clinicpathological parameters of NPC was analyzed as well. As shown in Table 1, PTEN CpG island with low methylation intensity was not related to age, gender, lymphatic node metastasis and tumor local grade. However, tumor local grade and clinical stages were significant difference between HM and UM groups.

**Table 1:** Association between PTEN methylation and clinicopathological parameters of NPC

	PTEN methylation						
	Total no.	M^c^		UM^c^	*p*^a^ value LM vs UM	*p*^a^ value HM vs UM	*p*^a^ value LM vs HM
		LM^c^	HM^c^				
Sex							
Male	39	14/39	17/39	8/39	NS	NS	NS
Female	11	5/11	4/11	2/11			
Age							
<60	37	15/37	15/37	7/37	NS	NS	NS
≥60	13	4/13	6/13	3/13			
T grade ^b^							
1, 2	33	14/33	10/33	9/33	NS	*p*<0.05	NS
3, 4	17	5/17	11/17	1/17			
N stage ^b^							
0	10	4/10	4/10	2/10	NS	NS	NS
1, 2, 3, 4	40	15/40	17/40	8/40			
Stage ^b^							
I, II	23	8/23	7/23	8/23	NS	*p*<0.05	NS
III, IV	27	11/27	14/27	2/27			
Histology							
Keratinizing squamous cell carcinoma	50	19/50	21/50	10/50			
Non-keratinizing carcinoma	0						

a Comparisons were made by Pearson Chi-square test or Fisher's exact test (SPSS 13).NS, no statistically different.

b Staging according to International Union Against Cancer (UICC).

c UM, unmethylated. LM, low methylation intensity. HM, high methylation intensity. M, methylated.

We further carried out IHC of PTEN expression in clinical NPC samples (Figure 2K). PTEN expression was lost in 27 of 50 tumor specimens of NPC, consisting of 15 in HM group, 10 in LM group, and 2 in UM group. The frequency of PTEN loss was significantly higher in HM group (71.4%; *p*<0.01) and LM group (52.6%; *p*<0.05) than that UM group (20%). This strongly supports that loss of PTEN expression is attributed to the higher intensity of PTEN promoter methylation upon the expression of EBV LMP1.

Collectively, these data suggest that EBV latent membrane proteins, particularly LMP1, may be involved in the higher methylation intensity at PTEN promoter, eventually resulting in the loss of PTEN expression.

### DNMT3b is involved in LMP1-induced high PTEN methylation intensity

**Figure 3:**
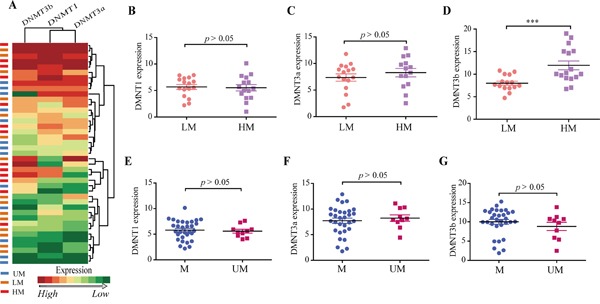
DNMT gene expression in different groups **A.** Hierarchical clustering of the DNMT1, DNMT3a, DNMT3b expression levels was generated by R programming language based on quantitative PCR results in NPC samples. High and low expressed genes are shown by red and green, respectively. **B-D.** Average expression levels of DNMT1, DNMT3a, and DNMT3b in LM and HM groups. **E-G.** Average expression levels of DNMT1, DNMT3a, and DNMT3b in M and UM groups. mRNA abundance was normalized to GAPDH mRNA. Each bar represents the means ± SD of three experiments. UM, unmethylated. M, methylated. LM, low methylation intensity. HM, high methylation intensity.

DNMTs control the methylation of cellular promoters [20]. To examine whether EBV LMP1-mediated DNA methylation is executed by DNMTs, we continued to detect DNMT gene expression profile in 50 NPC tissues (Figure 2B, 3A) and found that DNMT3b expression was more closely correlated with the methylation intensity at PTEN promoter than other two DNMTs (Figure 3A). The average expression level of DNMT3b but not DNMT1 and DNMT3a was significantly higher in HM group relative to LM group (Figure 3B-3D). We also compared the expression levels of three DNMTs between M and UM groups. As expected, no significant association of DNMT expression levels with the DNA methylation at PTEN CpG islands was observed (Figure 3E-3G). These results imply an important role of DNMT3b in facilitating a higher methylation intensity at PTEN CpG islands rather than initiating PTEN CpG island methylation.

**Figure 4:**
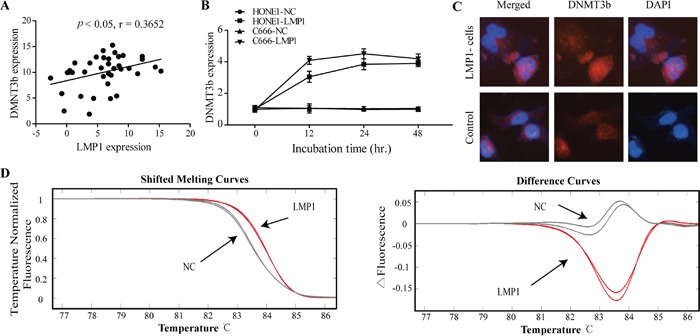
Activation of DNMT3b by EBV LMP1 induces the higher CpG island methylation intensity **A.** A statistically significant positive correlation between LMP1 and DNMT3b mRNA expression levels in NPC specimens (Spearman's correlation analysis, r =0.365, P < 0.05). **B.** Quantitative real-time analysis of DNMT3b in EBV-negative NPC cells (C666 and HONE1), induced by transient transfection of LMP1 gene. **C.** Immunofluorescence (400X) was done to visualize the expression and nuclear accumulation of DNMT3b between control and LMP1 gene-transfected NPC cells. **D.** HRM results of the control and LMP1 gene-transfected NPC cells. Melting curves of the methylated NPC tissues as well as the corresponding difference curves are shown.

Given that high methylation intensity at PTEN CpG islands is probably mediated by EBV LMP1, we further evaluated the correlation of DNMT3b expression with LMP1 expression in 40 NPC specimens with methylation at PTEN CpG islands. Statistical analysis revealed that the expression level of DNMT3b mRNA was positively correlated with LMP1 mRNA expression (2-tailed Spearman's correlation, r =0.365, P < 0.05) (Figure 4A). We also detected DNMT3b expression in LMP1-transiently transfected C666 and HONE1 cells (Figure 4B). After transfecting LMP1 into C666 cells, the mRNA expression level of DNMT3b gene was increased 4.05-fold at 12 hours and 4.2-fold at 24 hours, and sustained up to 48 hours. A similar phenomenon was observed in LMP1-transfected HONE1 cells (Figure 4B). Immuofluorescence analysis verified that DNMT3b was accumulated in the nuclei of NPC cells upon exogenic expression of LMP1 (Figure 4C). Moreover, we measured the PTEN CpG island methylation intensity in LMP1-transfected C666 and HONE1 cells. The shifted melting curves of MSP products amplified from LMP1 gene-transfected NPC cells were obviously higher than controls (Figure 4D).

Therefore, these results suggest that EBV LMP1 induces the high methylation intensity at PTEN promoter via activating DNMT3b.

### EBV LMP1 leads to the activation of DNMT3b via NF-κB signaling

**Figure 5:**
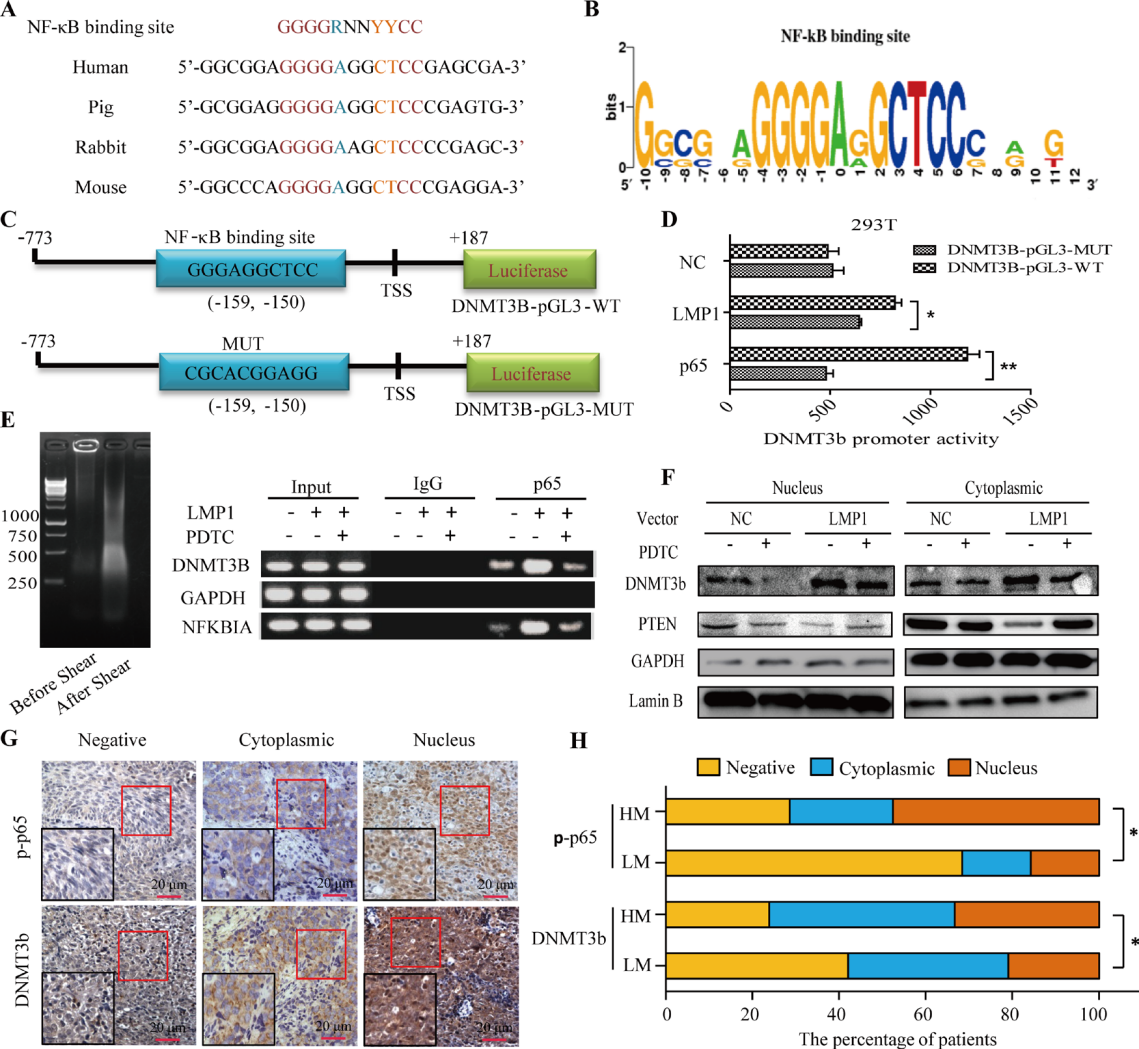
Activation of DNMT3b by EBV LMP1 involves NF-κB signaling **A.** Motif analysis of NF-κB binding sites in selected DNMT3b promoter sequences. Multiple sequence alignment among four species revealed a NF-κB binding site consensus sequence. **B.** Sequence logos depicting nucleotide distributions for the DNMT3b promoter NF-κB binding site. **C.** Schematic of the construction of the luciferase reporter vectors used to measure DNMT3b promoter activity. DNMT3b-pGL3-WT includes the complete 773 bp sequence directly upstream from the DNMT3b translational start site (TSS), and DNMT3b-pGL3-MUT includes the same sequence with the mutation of the putative B site. **D.** DNMT3b promoter activity assay. 293T cells were transfected with either DNMT3b-pGL3-WT or DNMT3b-pGL3-MUT alone or cotransfected with p65 or LMP1 vector. Luciferase activity was measured 48 h later. **E.** ChIP analysis of NF-κB promoter binding. C666 cells were either untreated, transfected with LMP1 gene alone, or transfected with LMP1 and a 5-h pretreatment with PDTC. The cells were harvested for ChIP assays using either IgG (negative control) or a p65-specific antibody. The primers used were specific for either the region of the DNMT3b promoter, including the κB site, the promoter region of the NFKBIA gene (positive control), or the promoter region of the GAPDH gene (GAPDH, negative control). **F.** Western blot analysis of DNMT3b proteins in cytoplasmic and nuclear upon exogenic expression of LMP1 alone, or a 5-h pretreatment with PDTC. **G.** Immunohistochemical analysis of p-p65 and DNMT3b in NPC tissues. **H.** The percentage of patients with the positive (nucleus/cytoplasmic) and negative expression of DNMT3b and p-p65 proteins in LM and HM groups. Each bar represents the means ± SD of three experiments. *p< 0.05, **p< 0.01. LM, low methylation intensity. HM, high methylation intensity.

To explore DNMT3b transcriptional regulation via its cis-regulatory elements, we scanned approximately 1 kb of DNA sequence located around the 5′ TSS of this gene using the prediction software TRANSFAC. A conserved putative NF-κB binding site (−159 to −150 bp) was found (Figure 5A and 5B). We performed Luciferase reporter assay in 293T cells transfected with a reporter plasmid containing DNMT3b promoter with a wild-type or a mutant form of NF-κB binding site (Figure 5C). Results showed that LMP1 gene-transfected reporter activities were higher in the wild type than in mutant cells (*p*< 0.05, Figure 5D).

NF-κB transcriptional factor is composed of two subunits, p65 and p50. To further test the possibility that NF-κB subunits directly regulated DNMT3b transcription by binding to DNMT3b promoter region, we performed ChIP assays and found that NF-κB p65 subunit constitutively bound to the DNMT3b promoter region, as indicated by the amplification of the promoter region encompassing the κB site from chromatin samples immunoprecipitated with p65 antibody but not IgG serum (Figure 5E). After 24-h LMP1 gene-transfection, the amount of p65 subunit bound to the DNMT3b promoter region was increased compared with basal levels (Figure 5E), which was similar to p65 binding to the promoter of NFKBIA, the gene encoding IκBα (positive control).

Furthermore, inhibition of LMP1-induced NF-κB activation by pretreatment with PDTC reduced the amount of p65 subunits binding to the DNMT3b promoter region to basal levels (Figure 5E). In consistence with the increase in DNMT3b protein level upon immunofluorescence analysis (Figure 4C), Western blot analysis displayed that DNMT3b was highly expressed and accumulated in the nuclei of NPC cells upon exogenic expression of LMP1. Notably, NF-κB inhibitor PDTC suppressed LMP1-induced DNMT3b activation (Figure 5F).

To characterize the in vivo correlation between the phosphorylation of NF-κB and the expression of DNMT3b, immunohistochemistry of p-NF-κB p65 and DNMT3b was applied to clinical NPC samples (Figure 5G). Notably, p-NF-κB p65 (+)/DNMT3b (+) cases were dominant (15 of 21, 71.4%) in HM group and relatively few (7 of 19, 36.8%) in LM group (P<0.05). p-NF-κB p65 and DNMT3b were more frequently localized in the nuclei of NPC cells in NPC cases of HM group (47.6% and 33%, respectively) relative to those from LM group (15.8% and 21.1%, respectively) (P<0.05) (Figure 5H). These results support that p-NF-κB p65 and DNMT3b overexpression were closely correlated in clinical NPC patients, particularly, those with HM at PTEN CpG islands.

Taken together, these data indicate that LMP1-induced NF-κB regulates DNMT3b at the transcriptional level through directly binding to a functional κB site on DNMT3b promoter.

## DISCUSSION

PTEN has been long believed to be important in controlling a wide range of physiological and cellular activities, such as metabolism and chromosome stability [1, 4, 21–23]. The loss of PTEN function is a major event in human carcinogenesis [1, 4]. Recently, we have preliminarily discovered the role of CpG island methylation in PTEN inactivation [5], but its molecular mechanism is still unknown. It is established that viral infections contribute to 15-20% of all human cancers [24, 25]. EBV, a human gamma herpes virus, is widespread throughout the world [26]. A reasonable hypothesis stemming from viral oncogenic mechanisms is that pathogens associated with the development of cancer may initiate or influence the epigenetic processes of host cells, leading to epigenetic reprogramming that represses or silences tumour suppressor genes [27, 28]. In this study, we demonstrate that EBV LMP1 can induce higher CpG island methylation intensity at PTEN promoter via NF-κB-mediated DNMT3b, highlighting a novel viral regulatory mechanism that contributes to the loss of PTEN function. Interestingly, recent investigations of potential EBV microRNA target genes revealed an inhibition of PTEN and extensive deregulation of several pathways involved in NPC. We previously reported that alteration of EBV-miR-BART1 and EBV-miR-BART7 expression results in migration and invasion of NPC cells through directly targeting tumour suppressor PTEN [29, 30]. Therefore, it suggests a possibility that aberrant CpG island methylation of PTEN confers “the second or additional hit” in NPC cells that contain EBV encoded microRNAs targeting this gene.

High PTEN CpG island methylation has been identified in various cancers including EBV negative subtypes [2, 31–34]. In the present study, conventional MSP assay also showed that PTEN promoter was methylated in both EBV-positive and EBV-negative NPC cells. No significant difference in the expression levels of EBNA1, LMP2A, and LMP1 was observed between methylated and unmethylated NPC tissue samples. This implies that EBV infection may be a necessary but not a sufficient factor for initiating PTEN CpG island methylation. Interestingly, using MS-HRM quantitating methylation intensity, we observed an increased methylation intensity of PTEN CpG islands in EBV-infected NPC cells, accompanied by decreased PTEN expression. To substantiate these observations in vivo, we further investigated the correlation between the expression of EBV genes and PTEN CpG-island methylation intensity in 50 NPC biopsies. There was no significant difference in the expression levels of EBNA1, LMP2A, and LMP1 between 40 methylated and 10 unmethylated NPC tissue samples, but LMP1 and LMP2A were significantly higher in high methylation intensity group than in low intensity group. These results collectively suggest that EBV-mediated the loss of PTEN expression may be attributed to the regulation of methylated PTEN CpG-island intensity rather than the initiation of PTEN CpG island methylation status in NPC cells.

To date, several mechanism models for the promoter methylation of host genes have been proposed based on the ability of EBV LMP1 to activate DNMT1 through various pathways [12–14, 35, 36]. For example, Siouda et al. reported that EBV LMP1 activated DNMT1 through activating E2F1/pRB signaling in EBV-infected primary human B-cells, resulting in the significant methylation of DOK1 promoter and expression silencing [13]. However, previous studies still ignored the establishment of DNA methylation and mammalian development mediated by the de novo methyltransferases DNMT3a and DNMT3b [37, 38], whereas DNMT1 just ensures the maintenance of methylation through replication [20]. Recently, DNMT3b has been closely linked to tumor development [39] and its expression and activity can be induced by EBV LMP1 [14, 36], but its underlying mechanism still needs to be clarified because de novo methyltransferases may silence specific genes in LMP1-mediated promoter methylation. In the present study, our experimental results support an important role of DNMT3b in facilitating a higher intensity of DNA methylation at PTEN CpG islands in the presence of EBV LMP1.

LMP1 is an EBV oncoprotein and identified as a viral “transforming” gene in early experiments done in rodent fibroblasts [40]. Several major signaling pathways associated with the C-terminal regions of LMP1 have been identified, including NF-κB, MAP kinase (ERK, p38, and JNK), and JAK/STAT [10, 11, 41]. In the present study, using the prediction based on Encyclopedia of DNA Elements at UCSC and the computational prediction for transcription factor-DNA binding sites [42], we discovered the NF-κB pathway potentially activated LMP1-mediated DNMT3b gene. Ultimately, we show for the first time that DNMT3b is a direct target of LMP1-mediated NF-κB signaling.

**Figure 6:**
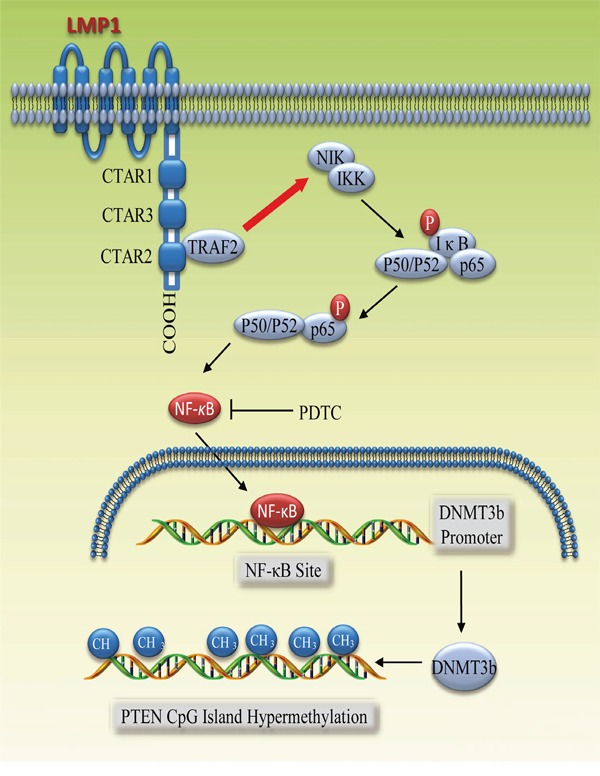
The model for LMP1-mediated DNMT3b activation via NF-κB signaling The CART2 domain of LMP1 activates the NF-κB signaling pathway, in turn, phosphorylates transcription factor p65. Phosphorylated p65 and p50/p52 complex binds and transactivates the DNMT3b promoter. Elevated DNMT3b expression eventually leads to higher CpG island methylation intensity of PTEN gene. This LMP1-mediated DNMT3b activation can be blocked by NF-κB inhibitor PDTC.

In conclusion, this study demonstrates that a latent viral protein activates the NF-κB signaling that enhances the expression of de novo methyltransferases DNMT3b, thereby leading to higher methylation intensity at PTEN CpG islands and eventually silencing major tumor suppressor PTEN (Figure 6). Conceivably, PTEN may not be the unique gene transcriptionally down-regulated by LMP1 via the NF-κB/DNMT3b signaling; it would be of interest to identify other genes that are potentially regulated by this similar mechanism. To date, the high CpG island methylation intensity of some tumor suppressors, RASSF1A, WIF1, DAPK1 and RARβ2, have been assessed by MS-HRM assays in NPC [43], so it is necessary to define whether DNA methylation of these promoters is also mediated by LMP1. Moreover, our findings further support that NF-κB and DNMT3b may be potential dual targets for treating EBV LMP1-positive tumors. Elucidation of this underlying mechanism is critical for the development of therapeutic agents.

## MATERIALS AND METHODS

### Ethics statement

All subjects involved in this study signed informed consent. The research was approved by the Ethics Committee of Southern Medical University, Guangzhou, China. The experimental protocol was established according to the associated national guidelines from Ministry of Science and Technology of China.

### Tissue specimens

All NPC (not pretreated with radiotherapy or chemotherapy) and non-cancerous nasopharyngeal (NP) specimens were collected and confirmed pathologically in the Department of Otorhinolaryngology, Nanfang hospital affiliated to Southern medical university, Guangzhou, China. DNA and RNA samples were collected from 50 NPC and 22 NP specimens for the bisulfite reaction and qPCR assay, respectively. The hematoxylin-and-eosin-stained frozen sections matched for each NPC tissue were examined to ensure that each tissue should contain more than 80% of homogeneous cancer cells in its cross-sectional area. For the use of these clinical materials for research purposes, prior written informed consent and ethics approval were obtained from all participants and the Ethics Committees of Nanfang hospital, respectively.

### Cell culture

Two EBV-negative NPC cell lines, C666 and HONE1, were obtained from Cancer research institute of Southern medical university, Guangzhou, China. Two EBV-positive NPC cell lines, C666-1-EBV and HONE1-EBV, were kindly offered by Professor George S. W. Tsao from the University of Hong Kong. All cell lines were cultured in RPMI-1640 (HyClone, Logan, UT, USA) with 10% calf serum (Gibco, Grand Island, NY, USA) at 37°C and 5% CO_2_.

### LMP1 expression vector and cell transfection

LMP1 vector was kindly offered by Professor Zeng Mu-sheng from Sun Yatsen university Cancer center, Guangzhou, China. Before transfection, the medium was changed to RPMI-1640 (HyClone) with 10% fetal bovine serum (Gibco). All cells were maintained in a humidified atmosphere of 95% air and 5% CO_2_ at 37°C, and seeded 24 hr prior to transfection. LMP1 vector and NC were transfected into cells at a final concentration of 50 nmol/l using Lipofectamine 2000 (Invitrogen, 11668-019) in serum-free conditions. Six hours later, the medium was changed to fresh RPMI-1640 (HyClone) with 10% fetal bovine serum (Gibco).

### Methylation-specific PCR

The methylation status of PTEN promoter region was determined by Methylation-specific PCR (MSP) using bisulfite-modified DNA. MSP assay was carried out on 8 CpG sites in its primer region. The sequences of the methylated primer pairs are shown in Supplementary Table 1 together with the other primer sequences used in the study. DNA was modified by the bisulfite reaction using an EpiTect Bisulfite kit (Qiagen). MSP experiments were performed at least in duplicate.

### RNA extraction and qPCR

Total RNA was extracted with TRIzol reagent (Invitrogen). cDNA was synthesized with the PrimeScript RT reagent Kit (TaKaRa, Dalian, China). PCR analyses were performed with SYBR Premix Ex Taq (TaKaRa). The primers used are shown in Supplementary Table 1. Data were normalized to GAPDH expression and further normalized to the negative control unless otherwise indicated. All reactions were run in triplicate and repeated in three independent experiments. The fold changes were calculated through relative quantification (2 ^−ΔΔCt^).

### Western blot analysis

Western blotting analyses were performed with standard methods. Briefly, cell pallets were lysed in the radio-immunoprecipitation assay buffer containing protease inhibitors (Sigma-Aldrich) and phosphatase inhibitors (Keygen, Nanjing, China). Proteins were separated by 10% SDS-PAGE gels, and blotted onto polyvinylidenedifluoride membrane (Millipore, Billerica, MA, USA). The membrane was probed with the specific antibodies (Supplementary Table 2), and then with peroxidase-conjugated secondary antibodies. GAPDH was used as a protein loading control. The bands were visualized by eECL Western Blot Kit (CWBIO Technology, Beijing, China). The images were captured with ChemiDocTM CRS+ Molecular Imager (Bio-Rad, Hercules, CA, USA).

### Methylation-sensitive high resolution melting (MS-HRM)

MS-HRM protocol was adapted from the method developed by Wojdacz et al [2, 3]. MS-HRM measures the fluorescence signal of an intercalating dye in double-stranded DNA. When the DNA melts, the dye is released and the intensity of the signal drops. In case of high methylation intensity DNA melts, there is an increased contribution of the unmethylated allele resulting in a relatively longer melting phase of the unmethylated allele, which results in a plateau phase at a lower fluorescence level. Similarly, low methylation intensity DNA melts results in a relatively small contribution of the unmethylated allele, leaving the plateau phase at higher fluorescence. HRM analysis was carried out on LightScanner System (Idaho Technology Inc.). The reaction volume was 20 μl and contained 10μl mix (Takara Code: DRR420A), DNA binding dye LC Green Plus, forward/reverse primers and 5 ng bisulfite modified DNA. PCR conditions and primer sequences are shown in Supplementary Table 1. The melting curves were normalized and calculated using the software provided with the LightScanner System.

### Dual luciferase assay

C666 cells (1 × 10^4^) were cultured in 24-well plates and co-transfected with 20 ng LMP1 vector or NC, 5 ng of pRL-CMV Renilla luciferase reporter and 30 ng of luciferase reporter that contained the wild-type (DNMT3b-pGL3-WT) or mutant (DNMT3b-pGL3-MUT) promoter of DNMT3b. Transfections were performed in duplicate and repeated in three independent experiments. Forty-eight hours after transfection, the luciferase activities were analyzed with a Dual-Luciferase Reporter Assay System (Promega, Madison, WI, USA).

### Immunofluorescent staining

Cell lines were cultured on coverslips, rinsed with phosphate-buffered saline after 24 h and fixed with 4% paraformaldehyde for 5 min at −20°C. The cells were next blocked for 30 min in 0.3% Txiton X-100, and then incubated with primary monoclonal antibody (DNMT3b) in phosphate-buffered saline for 2 h at room temperature. After three washes with phosphate-buffered saline, the coverslips were incubated for 1 h in the dark with secondary antibodies (Bioworld Technology, Inc, Nanjing, China). After further washing three times, the slides were stained with 4-6-diamidino-2-phenylindole for 5 min to visualize the nuclei, and viewed by confocal microscope (Olympus FV1000, Tokyo, Japan).

### Chromatin immunoprecipitation (ChIP) assays

ChIP analysis was run on three 10 cm plates per treatment of C666 cells using the EZ-ChIP™ Chromatin Immunoprecipitation Kit (Millipore, Catalog # 17-371) according to the manufacturer's protocol. A ChIP grade NF-κB p65 antibody used was purchased from Abcam, Inc. (Cambridge, MA, catalog no. ab2851). The IgG negative control antibody and the forward and reverse negative control GAPDH primers used were from the EZ-ChIP™ Chromatin Immunoprecipitation Kit. The sequences of the NFKBIA and DNMT3b primers are shown in Supplementary Table 1.

### Immunohistochemistry (IHC)

Paraffin sections prepared from in vivo experiments were applied to IHC staining for the detection of protein levels of PTEN and some key components of NF-κB/DNMT3b pathway. The indirect streptavidin-peroxidase method was used. All antibodies used for IHC were listed in Supplementary Table 2. The stained results were reviewed and scored by two pathologists independently.

### Statistical analysis

The data were expressed as the mean ± s.e.m. from at least three independent experiments. Comparisons between two groups were performed using Student's t-test unless otherwise indicated. The associations' among LMP1, LMP2A and DNMT3b gene were analyzed using Spearman's correlation coefficient. All statistical analyses were performed using the SPSS 13.0 statistical software package (SPSS Inc. Chicago, IL, USA). All statistical tests were two-sided, and p < 0.05 was considered to be statistically significant.

## SUPPLEMENTARY TABLES


