# Supplementary material for: Higher methylation intensity induced by EBV LMP1 via NF-κB/DNMT3b signaling contributes to silencing of PTEN gene
Source: Oncotarget. 2016 May 19;7(26):40025–37. doi: 10.18632/oncotarget.9474 (PMC5129989; doi:10.18632/oncotarget.9474)
Supplement: Supplementary file 1 [file oncotarget-07-40025-s001.pdf]

Higher methylation intensity induced by EBV LMP1 via NF-κB/ DNMT3b signaling contributes to silencing of PTEN gene

SUPPLEMENTARY TABLES

Supplementary Table S1: PCR primer sequences for qRT-PCR and Methylated specific PCR Assay

Primer	Sequence
qRT-PCR	
LMP1-forward	5’ TGGAGCCGGCGACGGA 3’
LMP1-reverse	5’ ACTGGGCCGTGGGGGTC 3’
LMP2A-forward	5’ TGCAATTTGCCTAACATGGA 3’
LMP2A-reverse	5’ GAGCACAAGCATCACCAGGA 3’
EBNA1-forward	5’ ACCGAGGATGGAAATTGGGT 3’
EBNA1-reverse	5’ CGCATCCTTCAAACCCTCAG 3’
DNMT1-forward	5’ CCCCTGAGCCCTACCGAAT 3’
DNMT1-reverse	5’ CTCGCTGGAGTGGACTTGTG 3’
DNMT3a-forward	5’ CCGATGCTGGGGACAAGAAT 3’
DNMT3a-reverse	5’ CCCGTCATCCACCAAGACAC 3’
DNMT3b-forward	5’ AGGGAAGACTCGATCCTCGTC 3’
DNMT3b-reverse	5’ GTGTGTAGCTTAGCAGACTGG 3’
GAPDH-forward	5’ CATGGGGTGTGAACCATGAGA 3’
GAPDH-reverse	5’ GTCTTCTGGGTGGCAGTGAT 3’
PTEN-forward	5’ TGCAGAAGAAGCCCCGCCA 3’
PTEN-reverse	5’ ACGCCTTCAAGTCTTTCTGCAGG 3’
Methylated specific PCR Assay	
Methylated	
PTEN-forward	5’ TTCGTTCGTCGTCGTCGTATTT 3’
PTEN-reverse	5’ GCCGCTTAACTCTAAACCGCAA 3’
Unmethylated	
PTEN-forward	5’ GTGTTGGTGGAGGTAGTTGTTT 3’
PTEN-reverse	5’ ACCACTTAACTCTAAACCACAACCA 3’
ChIP PCR Assay	
DNMT3B-ChIP-forward	5’ CTGGTTTTGGACGTCTGACC 3’
DNMT3B-ChIP-reverse	5’ GTGCCGACTCCCCTTGTAG 3’
NFKBIA-forward	5’ GACGACCCCAATTCAAATCG 3’
NFKBIA-reverse	5’ TCAGGCTCGGGGAATTTCC 3’

Supplementary Table S2: A list of antibodies used for Western blotting and IHC staining

Name of antibody	Cat. No	Company	Mol weight
PTEN	ab32199	Abcam	54 kDa
NF-kB p65	ab7970	Abcam	65 kDa
DNMT3b	ab13604	Abcam	110 kDa
GAPDH	P30008	Abmart	37 kDa
Lamin B	A2452	ABclone	66 kDa
